# Regional development potentials of Industry 4.0: Open data indicators of the Industry 4.0+ model

**DOI:** 10.1371/journal.pone.0250247

**Published:** 2021-04-19

**Authors:** Tímea Czvetkó, Gergely Honti, János Abonyi

**Affiliations:** 1 MTA-PE “Lendület” Complex Systems Monitoring Research Group, University of Pannonia, Veszprém, Hungary; 2 Institute of Advanced Studies Kőszeg, Kőszeg, Hungary; Universita degli Studi di Foggia, ITALY

## Abstract

This paper aims to identify the regional potential of Industry 4.0 (I4.0). Although the regional background of a company significantly determines how the concept of I4.0 can be introduced, the regional aspects of digital transformation are often neglected with regard to the analysis of I4.0 readiness. Based on the analysis of the I4.0 readiness models, the external regional success factors of the implementation of I4.0 solutions are determined. An I4.0+ (regional Industry 4.0) readiness model, a specific indicator system is developed to foster medium-term regional I4.0 readiness analysis and foresight planning. The indicator system is based on three types of data sources: (1) open governmental data; (2) alternative metrics like the number of I4.0-related publications and patent applications; and (3) the number of news stories related to economic and industrial development. The indicators are aggregated to the statistical regions (NUTS 2), and their relationships analyzed using the Sum of Ranking Differences (SRD) and Promethee II methods. The developed I4.0+ readiness index correlates with regional economic, innovation and competitiveness indexes, which indicates the importance of boosting regional I4.0 readiness.

## Introduction

In this rapidly changing environment, regions and cities are forced to develop their strengths to improve their overall competitiveness [1]. The challenge of today is to seize the opportunities hidden in digital transformation [2]. “Digital globalization is inducing deep and productive transformations, making industrial policy necessary in order to reorientate development towards inclusive and more sustainable growth” [3]. A major development potential of a region is managing information and knowledge flows between organizations [4]. Therefore, collaborative territorial governance has to take a step forward to help small and medium-sized companies apply Industry 4.0 and simultaneously develop the region itself [5]. Knowledge flow and innovation stimulates digital transformation by inter-regional cooperation projects while focusing on the green economy and smart cities concepts. This approach can result in the long-term sustainability of economic growth, reducing environmental risks and ecological degradation as well as improving human welfare and social equity [6]. “To support policymaking processes, it is necessary to develop analytical tools exploring the determinants of the Industry 4.0 development” [7].

This regional perspective and the concept of regional Industry 4.0 (referred to as Industry 4.0+) should gain more attention in the future as it opens up opportunities for regional developments that should be managed based on public and collective strategies. A decision support tool needs to be developed for I4.0+ projects to evaluate regional I4.0 performance. Recent studies have proven the potential of defining and measuring regional development status through indicator system assessment [7, 8]. Therefore, this paper aims to explore this potential and create a suitable tool to measure the I4.0 readiness of regions. A region-focused and I4.0-specific indicator system is built to provide a quantitative and comprehensively applicable concept. The developed indicator system is based on the Triple Helix model of innovation which refers that the synergies of governmental, academic, and business sectors amplify regional development [9]. The cross-collaboration of the Triple Helix actors enables a suitable and stable base for I4.0 implementation to be created and preserved. S1 Appendix underlines the importance of the Triple Helix concept by providing an overview of region-specific indicator systems utilizing the triple helix model.

In the following sections, the Industry 4.0+ (region-specific) concept and its major driving forces of development are discussed and analyzed. The Materials and Methods section defines the five major dimensions of the I4.0+ readiness concept and introduces the related open data indicators. Then, the possible application areas of the proposed I4.0+ index are determined. Furthermore, the methodology of developing and analyzing this regional readiness index is discussed in this section. The Results and Discussion section seeks to provide a clear and comprehensive analysis of the study applied to European NUTS 2 regions to measure their regional I4.0 readiness. The I4.0+ index was validated by correlation analysis using economic, innovation and competitiveness indexes.

## Materials and methods

The purpose of this chapter is threefold: 1) reveals the dimensions of I4.0-specific regional development, 2) identifies potential application areas and parties who may be interested in applying this concept, and 3) defines the methodological structure of the research (the development and analysis of the I4.0+ index and regional readiness ranking).

### Determination of the regional I4.0-related indicator system

This section reveals the necessity of measuring I4.0 readiness from a regional perspective. The core dimensions of the I4.0+ concept are determined and open data sources assigned to measure the I4.0 readiness of regions.

#### The dimensions of I4.0 development

The examination of the I4.0-related readiness models indicates a strong degree of organizational and national orientation. S2 Appendix provides an overview of the I4.0 readiness models describing the level of measurement and the areas of evaluation. Seven I4.0 readiness models are compared with regard to their scope and defined dimensions. According to the findings, five out of the seven studies defined readiness models for companies (mainly SMEs) ([10–14]), while one each is designed for the national [15] and city levels [16]. However, recent studies have tended to pay increasing attention to the perspective of regional development in the case of I4.0. Ref. [8] developed a Regional Industry Index (RII) based on ten open data indicators applied for Bulgarian NUTS 3 regions. The study highlighted the role of the labour market, investments, enterprises, R&D and information society. Ref. [7] seeks to explore the determinants of I4.0 development using extended indicator-based SWOT analysis applied to a Polish region (Podlaskie Voivodeship). Furthermore, Ref. [17] defines the correlation between Industry 4.0 and R&D projects in relation to stages of the macroeconomic cycle. However, it can stil be claimed that the regional assessment of I4.0 readiness has not been studied extensively. Furthermore, regional readiness significantly determines the development status of both organizations and nations. Regions should sufficiently combine regional resources with new industries and technologies to avoid the efficiency trap in terms of technological innovation while focusing on industrial transformation and creating industrial opportunities [18].

**Fig 1:**
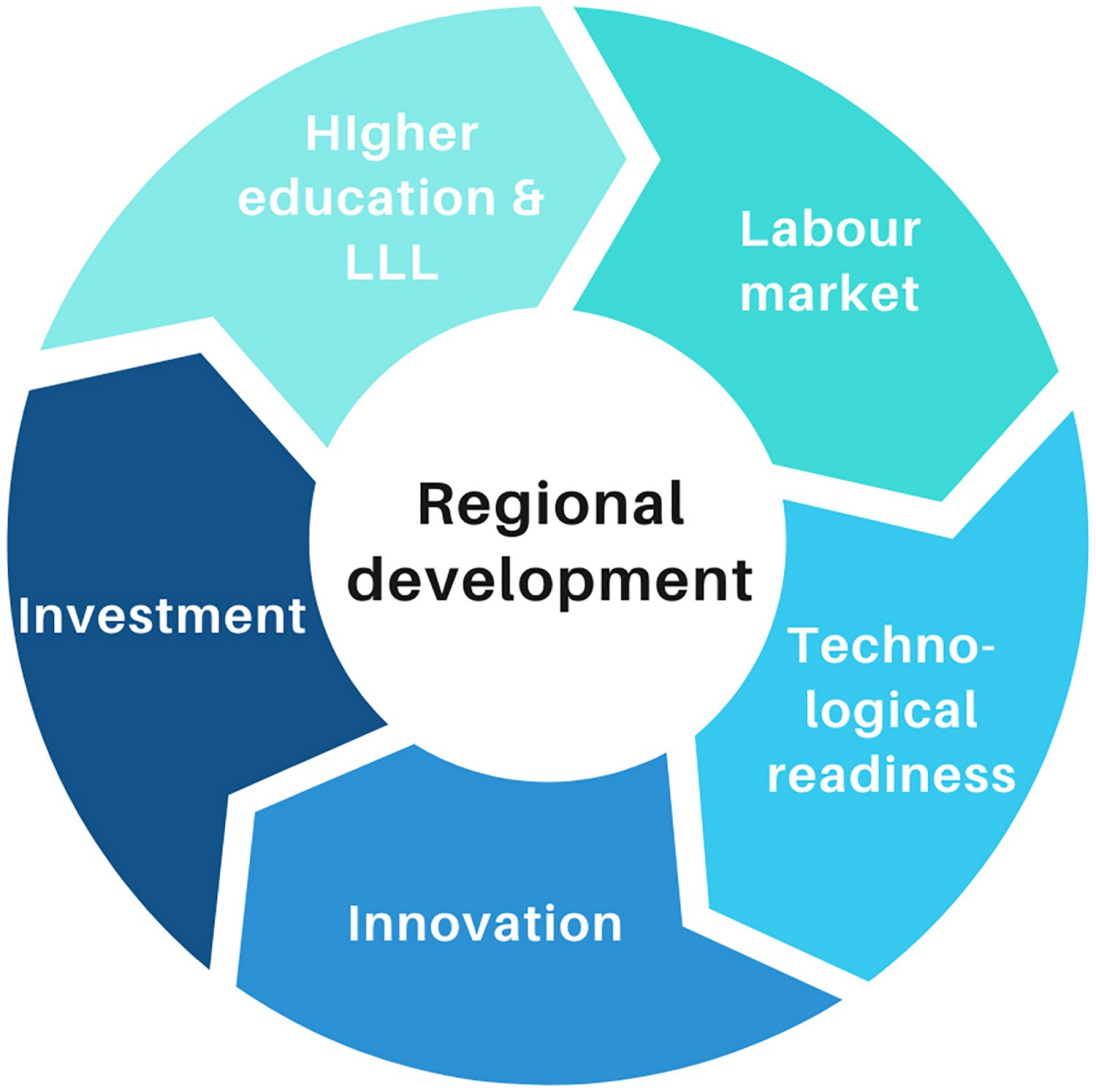
I4.0-related dimensions of regional development.

Even though the analysis of Refs. [7, 8] determines the regional development potential in a well structured way, the NUTS 2-classified and multi-source data analyses are still missing. Therefore, we see the potential in developing a solely open data indicator-based I4.0+ index to measure the NUTS 2 regional readiness for I4.0 implementation and, as a result support decision-making processes. The developed I4.0+ indicator system is divided into five major dimensions representing regional economic growth: higher education and lifelong learning, technology, innovation, investment and the labour market. The dimensions mentioned in Fig 1 are strongly related to each other and aim to foster regional development and competitiveness and, therefore, improve the quality of life.

These dimensions are interrelated to the Triple Helix actors, academia (universities and research institutions), industry (organizations and the labour market) and the government. Higher education is like a driving force of knowledge creation, providing a well-educated workforce for the labour market. Moreover, this sector also supports innovative actors, such as research institutions. The innovative capability of a region can redound to the ability to apply modern-day technological developments. On the other hand, investment is essential to promote knowledge transfer. However, projects may not be implemented by enterprises or institutions without financial support. The willingness to continually learn policies can be the basis for successful scientific and technological activities [19]. The socio-technical environment integrates and shapes the governance of innovations and transformation processes [20]. All these dimensions aim to enhance the competitiveness and development of a region. In the following, these dimensions are analyzed more in-depth, and the indicators associated with them proposed.

#### Higher education and lifelong learning

Due to constant digital transformation and innovation, the demand for an immensely skilled workforce with new skill sets is increasing [21]. Regarding the expansion of technology-based knowledge and the advent of the global economy, education is directly responsible for the evolution of industrial structures [22]. Therefore, the importance of on-demand educational training and lifelong learning activities is beyond doubt as the reskilling of the workforce facilitates the ability of people to cope with future job creation. As members of the academic sector, universities are said to be one of the drivers of regional economic development and competitiveness. They play a significant role in rebounding socio-economic advancement through studies, education, innovation and infrastructure development [23].

Regarding the dimension of regional higher education and lifelong learning, the following indicators are considered:

- Educational attainment level (25-64) (EDU1-4) determines the percentage of the population aged 25-64 that falls into different levels of educational attainment. Three levels of educational attainment are considered, namely 1) upper secondary as well as post-secondary non-tertiary and tertiary education, 2) upper secondary as well as post-secondary non-tertiary education, and 3) tertiary education. Therefore, by measuring the percentage of 25-64 aged population attainment in terms of higher education, this indicator can be considered to identify the intensity of lifelong learning within regions.
- The educational attainment level (30-34) (EDU5-7) offers a narrower perspective of the aforementioned indicator as the age group is only between 30 and 34 years old. It measures the percentage of attainment of the population in terms of educational level.
- The indicator of the employment rate of young people not in education and training (aged 15-34) (EDU8-35) aims to measure the gap in time between when young people finish education and enter the labour market.
- Graduates (BSc, MSc, PhD) in Engineering, the Natural Sciences and Statistics (GRA1); IT and Information and Communications Technology (GRA2); as well as Engineering, Manufacturing and Construction (GRA3) can be some of the significant forces with regard to the I4.0-related pillar of higher education. This indicator determines the regional preparedness of providing labour to the market that is specifically skilled in I4.0. Furthermore, this output indicator can be considered from the perspective of the demand for skilled labour of regions with regard to I4.0.
- The number of students participating in study mobility programmes (ERA1-2) indicates the additional number of students leaving and arriving in a region to study in the fields of Science, Mathematics and Computing, as well as Engineering, Manufacturing and Construction.

#### Labour market

Opportunities as a result of Industry 4.0 made developing businesses conscious as continuous innovation is no longer an option for success but necessary to remain competitive in the market. Careful and conscious business innovation has become a priority, which has changed the perception of job content. As a result of digitalization and robotization, there is a growing need for high technology and knowledge-intensive services. According to a report from the World Economic Forum (WEF), 23 to 37% of companies are planning to invest in robotization between 2018 and 2022. This transformation brings about 133 million new jobs and help to balance out almost 75 million jobs displacements [24]. Governments and institutions face the pressing issue of identifying skill development models to ensure job opportunities for the working age population [25].

Three indicators are considered to identify the readiness of regions with regard to the I4.0-related labour market, namely:

- Employment in the technology and knowledge-intensive sectors (HTE1-8) reflects the percentage of employment in the chosen economic activities related to I4.0.
- The number and tone of I4.0-related media appearances (GDC, GDT) within regions is a creative way of measuring how many regions deal with the issues and/or results of the relevant field (innovation, automation, research and development as well as SMEs).
- The number of institutions (GRC) within a region indicates the available employment opportunities as well as the possibility for open innovation and collaboration with external parties. Within this indicator, we consider research-related organisations registered in the Global Research Identifier Database [26], namely educational institutions, companies, healthcare institutions, governmental institutions and nonprofit organization.

#### Innovation

The innovation capability of a region is a critical factor in terms of I4.0 readiness, as it empowers a company or region to be competitive [21]. Innovation can occur through the utilization of technological developments as well as idea and knowledge creation [27], in which the Triple Helix actors play a significant role [28]. Their collaboration increases the efficiency of finding solutions to innovation and sustainability-related challenges [29].

The following indicators are taken into account with regard to the I4.0-related innovative actions of regions, namely:

- The indicator of Human Resources in Science and Technology (HRS1-10) reflects the regional intensity of employment in science and technology. It determines which regions are advanced and which need to improve.
- Patent applications in I4.0-related field (PAT) indicates the innovation capability according to the ability and willingness of a region to adopt new ideas and technological developments.
  These I4.0-specific areas are the following: Additive manufacturing technology, Nanotechnology, Machines or engines in general; Engine plants in general; Steam engines, Controlling; Regulating, Computing; Calculating, Counting, Signaling, Information and Communication Technology (ICT) especially adapted to specific fields of application.
- Publications by category (PAP) aims to measure the number of publications per region over a given periof of time. This indicator reflects the intensity of research and development actions in the academic sector.
  These categories are the following: Industrial engineering, Operations management, Process engineering, Transport engineering, Operations research, Simulation, Knowledge management, Control theory, Telecommunications, Mechanical engineering, Computer engineering, Software engineering, Manufacturing engineering, Machine learning, Data mining, Mathematical optimization, Control engineering, Regional science, Embedded systems, Artificial intelligence, Process management, Reliability engineering, Systems engineering, Management science, Data science.
  A strong correlation can be observed between productivity indicators with regard to the number of publications and university-industry co-authored publications that exist [30].
- Employment in the technology and knowledge-intensive sectors (HTE1-8) is a driver of innovative progression as employees in these sectors are the “initiator” of knowledge transfer and development.

The dimension of innovation interweaves all sectors that are considered drivers of regional I4.0 development, namely higher education and lifelong learning, the labour market and technology, as well as investment.

#### Investment

Financial initiatives play a significant role in terms of skill evolution, innovation and job creation through policy-making. The industry has been a target of investments as it is expected to be a driver of competitiveness, innovation, jobs and wealth [31]. The responsibility of the budget allocation for R&D must be divided among the Triple Helix actors [28]. Furthermore, I4.0-focused regional development can only be maintained with collaboration between the actors (academia—key of knowledge, industry—production key, government—key of stable interaction).

Both R&D personnel and R&D expenditures considered to be elements of innovation with regard to the regional innovation system [32], therefore, the dimension of I4.0-related investment studies the following two indicators:

- Total intramural R&D expenditure (GERD—Gross domestic expenditure on R&D) (RDE1-5) indicates the source of funds for research and development within a region by sectors. This indicator determines the density of financial support with regard to R&D.
- The total R&D personnel and researchers (RDP1-15) indicator reflects individuals employed in the research and development field as well as researchers employed in the public or private sectors as academia. This indicator enables knowledge workers considered to be drivers of knowledge, innovation and facilitators of improvement to be identified.

#### Technology

“The successful adoption of the 4th industrial revolution will rely on the ability of governments, business and citizens to commit in supporting the transformation of society into a modern and smart society driven by advanced technology, skills, innovation and responsive policy” [21]. Technological diversification can promote regional innovation capability [33].

To identify the technological readiness of regions, the equipment of businesses alone cannot determine the suitable environment for adapting and utilizing emerging technologies. A high degree of flexibility is essential to provide a basis for digital culture and skills [34].

“Networking with external institutions highlights R&D collaboration of universities or public research institutions with industries, e.g., high-tech ventures or small-sized enterprises to foster technology convergence. Meanwhile, governmental supports can play a catalytic role in stimulating technology convergence networks” [35]. Spatial interconnections can be exploited by external parties such as universities, companies or the government, leading to an improved and competitive environment.

In this regard, the technological factors affecting regional I4.0 readiness are the following:

- Employment in the technology and knowledge-intensive sectors (HTE1-8) can reflect the capability to adapt new technologies and the constant demand for modernization.
- The output of I4.0-related publications (PAP) indicates the intention to deal with modern-day technologies and future challenges.
- Patent applications (PAT) in the fields of Additive manufacturing technology, Nanotechnology, Machines or engines in general; Engine plants in general; Steam engines, Controlling; Regulating, Computing; Calculating, Counting, Signaling, and Information and Communication Technology (ICT) reflect the regional capability with regard to the adaption of new technologies.
- The indicator of Graduates in the fields of IT, Engineering or Mathematics (BSc, MSc, PhD) (GRA2-3) assumes the need for I4.0-related skills and graduates can be considered as the basis of knowledge transfer for an innovative technology-oriented environment.

### Application areas of the proposed I4.0+ index

**Fig 2:**
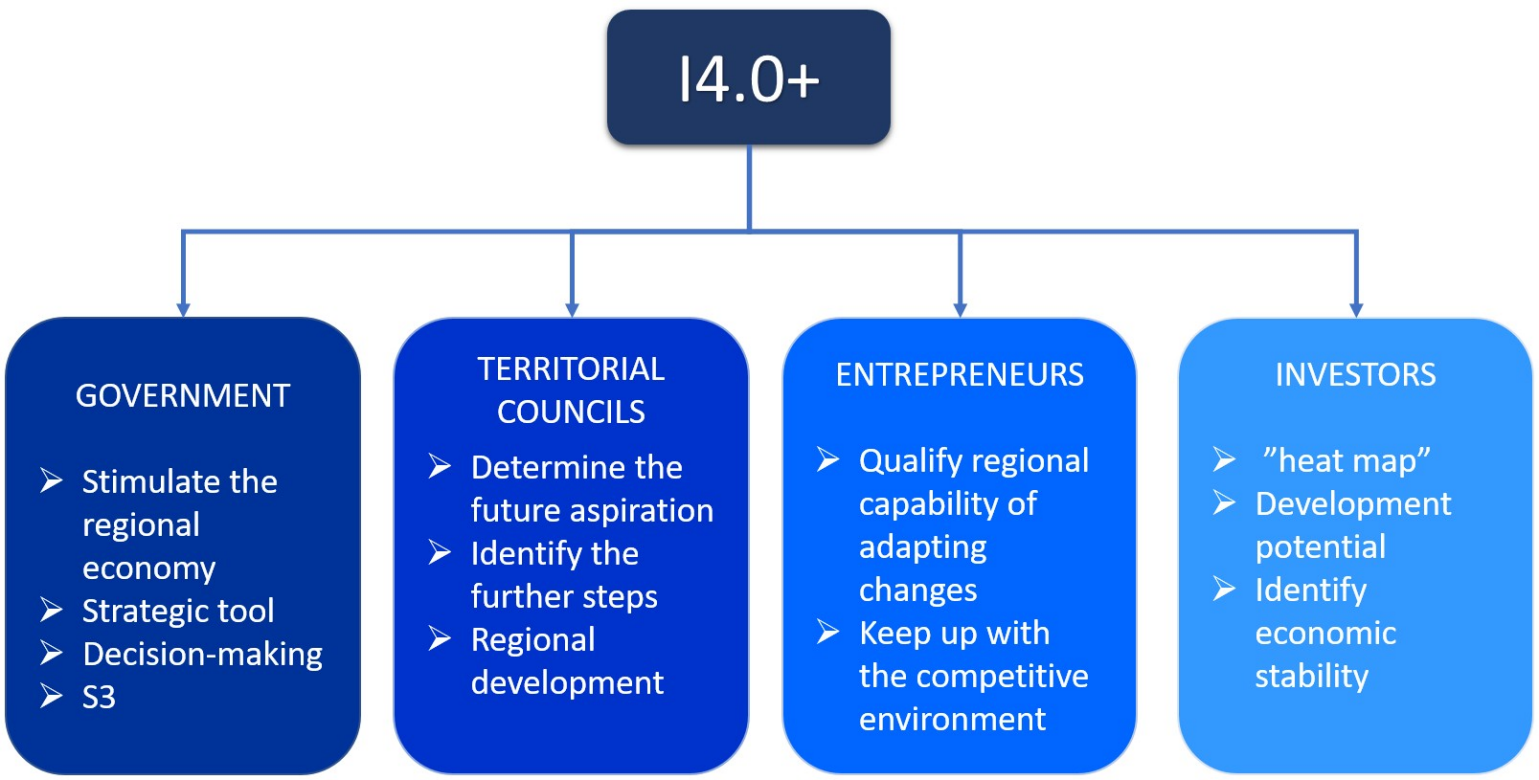
Possible areas of application of the I4.0+ index to utilize the potential in terms of regional strength.

Identifying the stakeholders and the applicability of the proposed index is critical as the possible future demand and interest must be considered. We classified four possible parties, which are shown in Fig 2, who could benefit from utilizing the I4.0+ regional readiness index and conclude the regional development status.

1. Governments have power over regions not just financially but in terms of policy-making processes as well. By considering regional economic development, governments can use this index to measure regional statuses and identify future strategic plans for their improvement. This can include research-and-development allocations, innovation projects and Smart Specialization Strategies (S3) between regions. If a country wants to make its mark on the “global world map” it has to start developing from lower levels and keep up with continually changing global requirements.
2. The role of territorial councils is also appreciated as they are directly involved and enable to identify what has to be improve. The I4.0+ readiness index confirms the position of regions in the ranking. Regions can determine their future aspirations and further steps in order to be competitive regionally and strong economically. The innovation-helix is performed in the regional stage and can create connections between businesses, governments and academia.
3. Entrepreneurs connected to the business sector can use the index as a tool for identifying the capability of regions to remain or become stable. They can look into which regions are worth investing in and how regions can develop by the concept of I4.0. The connection of enterprises to this concept is critical as they can function as the potential drivers of economic growth.
4. Investors can use the proposed indicator system as a “heat map” to determine which region(s) is worth investing in, or stable enough to cope with future challenges. The area of investment is critical in terms of research and development, manufacturing or attracting businesses to a region.

### Development and analysis methodology of the I4.0+ regional readiness index

The major objective of this section is to provide a methodological workflow for developing and analyzing the I4.0+ index. The reader is guided through the process of selecting relevant data metrics and its analysis to derive an I4.0 and NUTS 2-specific indicator system capable of ranking regions according to their I4.0 readiness.

**Fig 3:**
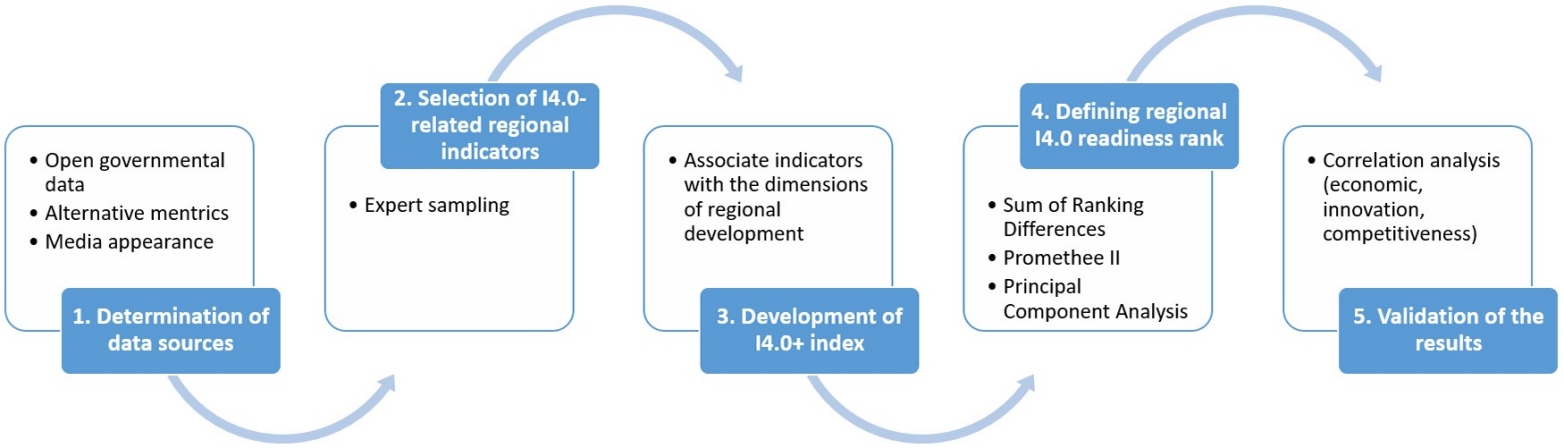
The methodological workflow of developing and analysing the I4.0+ readiness index.

Fig 3 provides an outline of the methodological steps, which are individually discussed below:

1. **The determination of data sources and I4.0-related indicators to measure regional readiness**
  The following types of data sources were considered in terms of the development of a dataset for measuring the I4.0 readiness of NUTS 2 regions:
  (a) *Open governmental data*: Eurostat Regional statistics
  (b) *Alternative metrics*: Beyond open governmental data, reports and open data platforms were used in order to broaden the scope of examination in the field of I4.0. Table 1 shows the sources and connected indicators determined along with the spatio-temporal horizon.
  (c) *Media appearances*: The GDELT Project [36] makes it possible to overcome the spatio-temporal boundaries by media appearances. To precisely examine the appearance of I4.0-related news stories, we concentrated on four main topics, namely innovation, automation, research and development, and SMEs. In this regard, the following search words and areas were examined: Jobs, Competitive industry, Education skills development and labour market, Industry policy, Employability skills and jobs, Manufacturing
  Detailed descriptions of the identified variables are shown in S3 Appendix.
  Table 1 shows the analyzed reports and open data portals considered through the selection of possible indicators describing regional I4.0 readiness.
2. **Selection of I4.0-specific NUTS 2 regional indicators—the development of the I4.0+ readiness index**
  With regard to the previous step of identifying relevant data sources, this step concerns the selection of indicators able to describe regional readiness.
  The data selection occurred through expert sampling.
  As a final set of data sources, seven platforms are chosen and associated with the five dimensions of I4.0+ as follows:
  (a) Higher education and lifelong learning: Eurostat Regional statistics, ETER (European Tertiary Education Register), Erasmus+ Study Mobility Program, Global Research Identifier Database (GRID);
  (b) Labour market: Eurostat Regional statistics, Global Research Identifier Database (GRID), The GDELT Project;
  (c) Innovation activities: Eurostat Regional statistics, Microsoft Academic Graph (MA-Graph), United States Patent and Trademark Office (USPTO);
  (d) Investment: Eurostat Regional statistics
  (e) Technology: Eurostat Regional statistics, ETER (European Tertiary Education Register), United States Patent and Trademark Office (USPTO);
  Regarding the expert sampling, 101 indicators were selected that describes NUTS 2 regional I4.0 readiness. A detailed description of the data describing the regional Industry 4.0 readiness index is found in S3 Appendix.
  The I4.0+ indicator system is based on the five dimensions of regional development of I4.0 mentioned in Fig 1. The dimensions of the indicator system are weighted equally.
3. **Determination of the ranking of NUTS 2 regions regarding their I4.0 readiness**
  The ranking of regions according to their I4.0 readiness is based on the following methods:
  (a) Indicators were analyzed using the **Sum of Ranking Differences (SRD)** method [45] that compares variables based on the Manhattan distance between the sum ranked item differences in case the gold standard is fixed for the comparison. This method identifies and ranks indicators regarding their ability to describe the topic and reveal indicators ranked reverse. The results are visualized in two-dimensional space.
  (b) The **Promethee II** method [46] is applied to the indicator system that provides a pairwise comparison of the criteria. The **Promethee- GAIA** refers to a k-dimensional visualization of the set, which provides a more efficient representation.
  (c) **Principal Component Analysis (PCA)** [47] provides a linear combination of the variables which is a widely used technique for data processing and dimensionality reduction. The biplot of PCA shows the alternatives, in this case, a two-dimensional space determined by the principal components.
4. **Validation of the results**
  As a result, an I4.0+ index is provided, which ranks regions regarding their I4.0 readiness. To validate the results, the correlation between the I4.0+ index and the economic (GDP), innovation (RII—Regional Innovation Index), and competitiveness (RCI—Regional Competitiveness Index) indexes is determined.

**Table 1:** Analysed reports and open data portals as data sources.

Source	Type	Examined indicator/data	Time horizon	Spatial horizon
Regional Innovation Scoreboard (RIS) [37]	Report	Innovative SMEs collaborating with others as a percentage of SMEs	NUTS 1 and 2 for different countries for CIS 2008, CIS 2010, CIS 2012, CIS 2014, CIS 2016	NUTS 1 and 2 for different countries for CIS 2008, CIS 2010, CIS 2012, CIS 2014, CIS 2016
Regional Competitiveness Index (RCI) [38]	Report	Knowledge workers (% of total employment)	average 2015-2017	NUTS 2
Cultural and Creative Cities Index (C3) [39]	Report	Average appearances in university rankings	2018	City
ETER [40]	Open data portal	Graduates in IT, Engineering and Mathematics (BSc, MSc, PhD)	2008-2016	NUTS 2
Erasmus+ [41]	Open data portal	Number of students participating in mobility programmes	2008-2013	NUTS 2
MA-Graph [42]	Open data portal	Publications by categories	2008-2018	NUTS 2
GRID [26]	Open data portal	Distribution of institutions by categories	-	NUTS 2
USPTO [43]	Open data portal	I4.0-related patent applications at the NUTS 2 level	2008-2018	NUTS 2
CORDIS [44]	Open data portal	Collaboration between organizations under Horizon 2020	2014-2020	NUTS 2

## Results and discussion

### Regional ranking according to their I4.0 readiness

This section refers to the regional I4.0 readiness ranking methodology and the analysis of the given results. Indicators were analyzed using both the Sum of Ranking Differences (SRD) and the Promethee II method. The ranking of regions is interpreted in a two-dimensional space based on the Principal Component Analysis (PCA) method.

Promethee II is a multicriteria decision aid method based on preference ranking organization, while the Promethee-Gaia method visualizes the set in a k-dimensional space [46]. SRD compares variables based on the Manhattan distance from the gold standard [45]. The similarities between the examined indicators were evaluated by the SRD method [45]. SRD is an effective way to analyze which indicators best describe our concept or are more backward and ranked in reverse. The PCA method is used to reduce dimensionality, and the ranking of alternatives (NUTS 2 regions) is presented in a two-dimensional space determined by the principal components (PCs). The principal components define which variables describing the topic the most (PCs are the linear combination of the variables).

**Fig 4:**
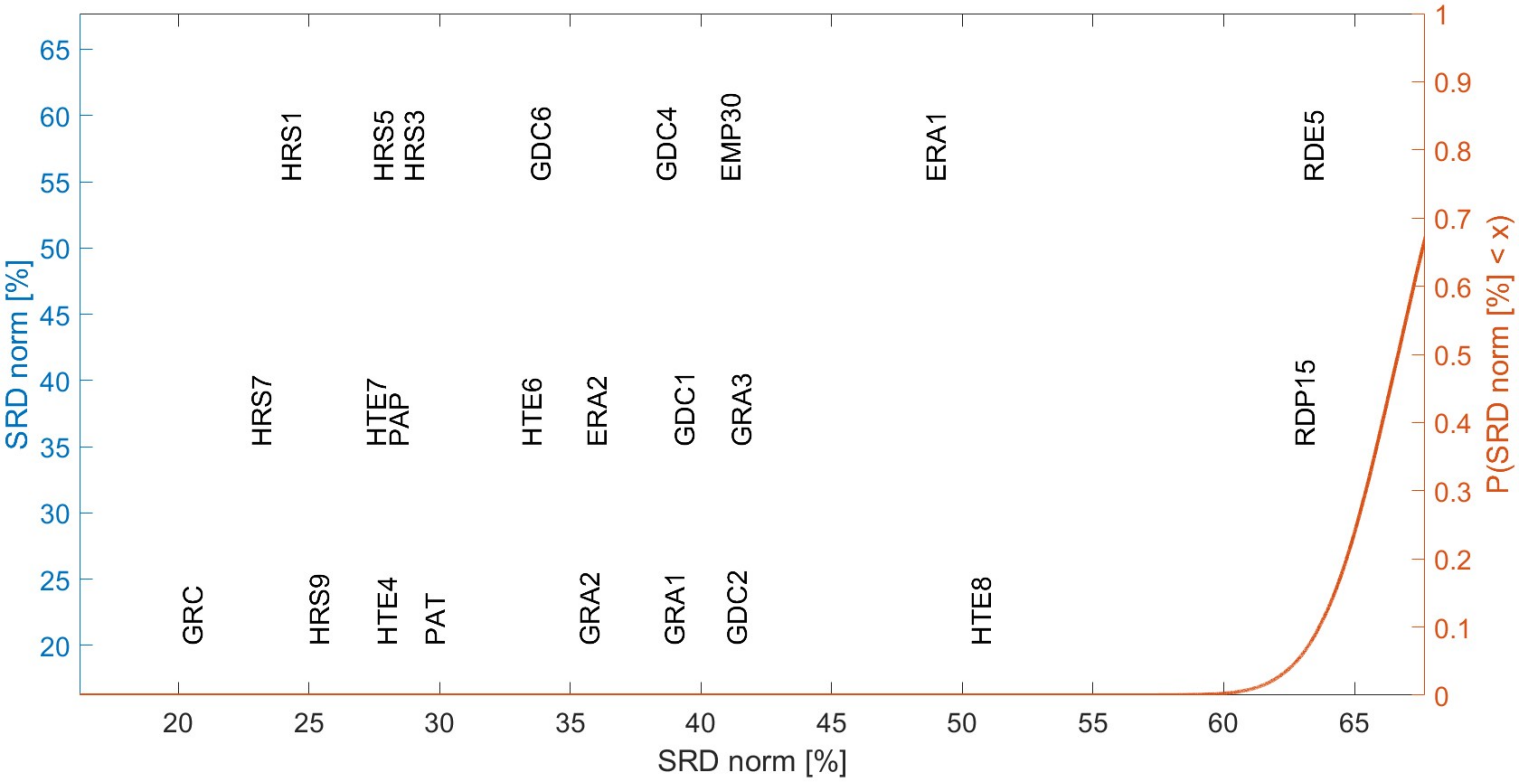
Selected variables from SRD according to their relevance in measurement. (The descriptions of variables are shown in S3 Appendix).

The selected variables are shown in Fig 4 which reflects the main components describing I4.0 readiness. It is worth noting the two significant groupings, namely indicators connected to employment as well as the research and innovation-oriented indicators.

The former indicates the importance and connection between education and innovation as there is an increasing need for a qualified workforce and scientific activities.

1 Total Employment rates of young people not in education and training (EMP30): This includes all educational levels and education between the ages of 15 and 34. It measures the number of people employed after finishing their education and categorizes them according to the years passed since completing their highest level of education.
  The analysis of this indicator concerning the complex nature of employability and job opportunities can be a first step to reflect how difficult it is to get a job (measured in years). It reflects the development stage of an economy. We can assume where the employment rates of young people are higher (after finishing their education less then 2-5 years), job opportunities exist and economic value is generated in a region which fosters adaptation capability. However, it must be noted that other factors also shape this aspect.
2 The number of graduates in I4.0-related fields (GRA1, GRA2, GRA3): The I4.0-related educational programmes which are in demand that foster students (future workforce) to build an innovative mindset and gain I4.0-relevant skill sets.
  It should be noted that the Number of students participating in mobility programmes in the field (ERA2) is closely related to graduates.
3 The number of news concerning ‘competitive industries’ (GDC1), and ‘education skills development and labour market’ (GDC2) also appears. The number of news stories found can be considered to be a soft indicator for measuring the public awareness of the field, which can further be analyzed with regard to the tone of the news (attitude towards the events; whether it is positive, negative or neutral).

The second significant group is the research and innovation area, which correlates with labour, educational and technological factors. This is underlined by indicators concerning people employed in science and technology as well as the employment rate in the high-technology sector. Furthermore, investment also plays a significant role in the research and innovation area.

4 Human Resources in Science and Technology under the category of Persons employed in science and technology (HRS7) is the following indicator which is the closest to measure excellence in the field. It measures the percentage of the total population employed in science and technology as well as reflects the emerging need for researchers and scientific actions.
5 The density of research institutions (GRC) is another relevant variable, as it measures the number of institutions involved in research activities.
6 Human Resources in Science and Technology in the category of Scientists and engineers. As has previously been mentioned, the employment rate in science and technology plays an essential role in terms of development. This indicator is one of its sub-indicators, so its relevance is highlighted even more.
7 Employment in High-technology sectors (high-technology manufacturing and knowledge-intensive high-technology services) (HTE4, HTE7) measures people employed in this field as a percentage of the total employment. It indicates the demand of the labour market in the field, reflecting how regions keep up with advanced technologies.
8 Industry 4.0-related publications (PAP) are also heading in the direction of research activities and indicate regional maturity with regard to how much a given region is involved in I4.0 (research) projects.
9 Industry 4.0-related patent applications (PAT) approximate the direction of the Human resources in science and technology indicator. It is unequivocal that they are strongly correlated as those employed in science and technology are the reason for the density of patent applications (effects) within a region. This indicator can identify the research activities and innovation capabilities of a region.
10 The appearance of Industry 4.0-related news is located over one-third of the scale as is the case quite close to the reference. I4.0-related media appearances can from one hand qualify the events that have occurred in the relevant field, but on the other hand PR activities influence the number of news stories that occur in a region.
  It is fascinating that Intramural R&D expenditures (GERD) are located on the same scale as news appearances. Consequently, we can claim that GDELT can function as a “proxy” indicator and assume how much is spent on research and development actions in each sector. Furthermore, it should be noted that according to the Eurostat database, the latest available data on GERD is from 2016, while the available news in GDELT is continuously updated.
  On the other hand, while the number of news has a significant effect, its tone does not provide added value for the index.

**Fig 5:**
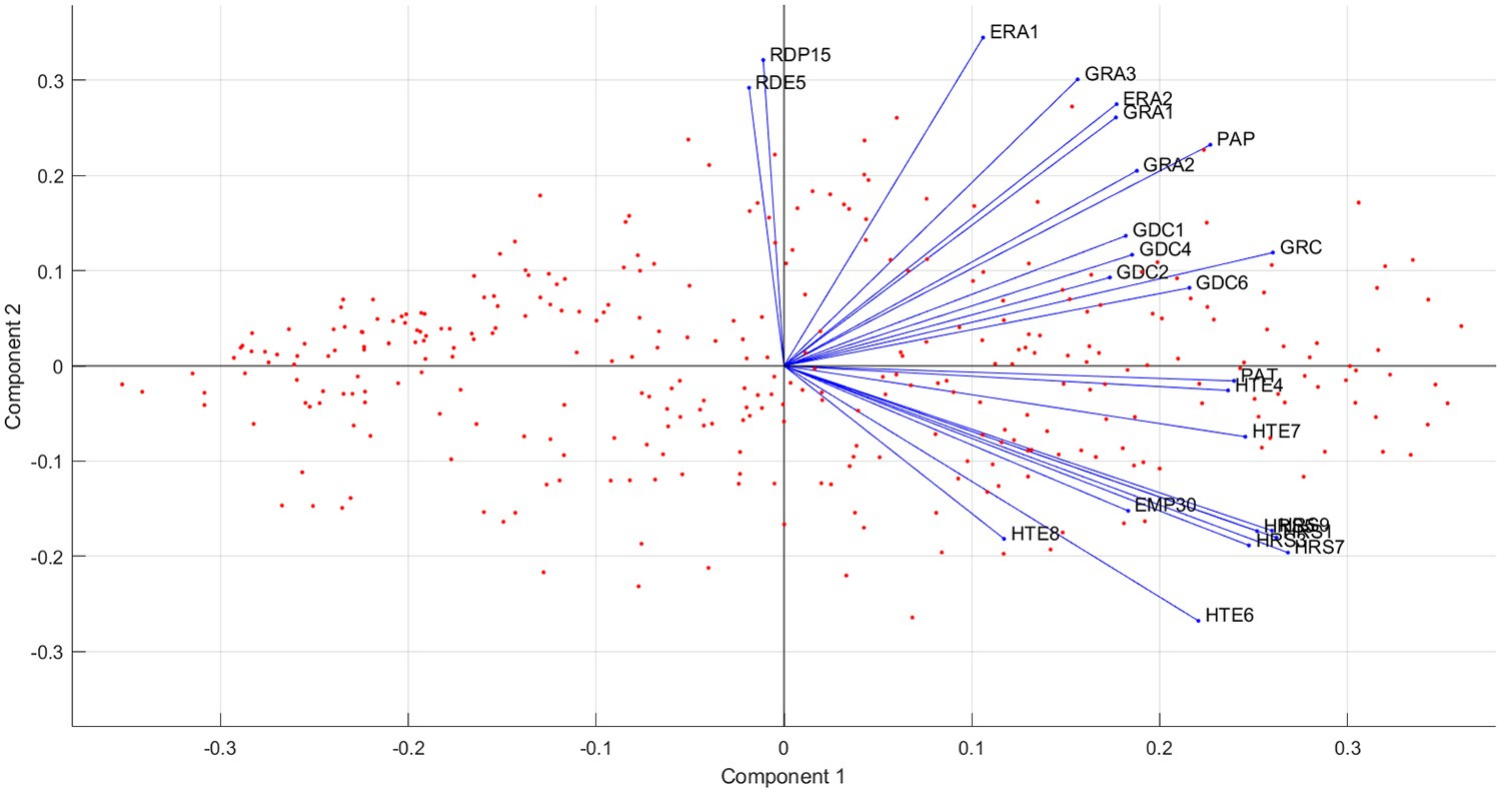
Selected factors according to their importance measured by promeethe-GAIA.

Fig 5 shows the ranking according to the Promethee method [46], as it makes a pairwise comparison according to the criteria and creates the full or partial ranking of alternatives. Visualization takes place by Promethee-Gaia method, while the ranking of variables is interpreted by the Principal Component Analysis of decision-making processes (preferences) calculated by criteria. Likewise, indicators that point the furthest, in the same way, are the most determinative.

The presented results underline the outcome of the SRD method, which categorized the two leading groups of indicators as (1) employment and (2) research and innovation actions. In this case, these factors are the Human Resources in Science and Technology (HRST), Total Employment rates of young people not in education and training (EMP), Employment in technology and knowledge-intensive sectors (HTE) and the Total R&D personnel and researchers (RDP).

**Fig 6:**
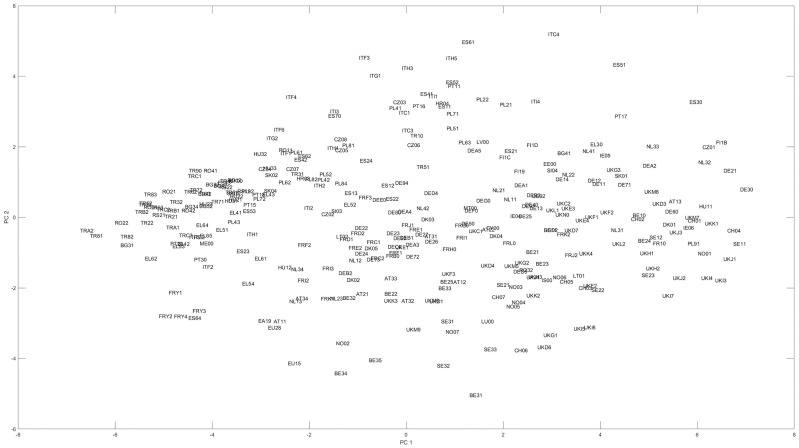
The layout of the regions according to the PCA method indicates regional readiness [48].

After analyzing the indicators, the ranking of regions is presented in a two-dimensional space, based on Principal Component Analysis (PCA). The horizontal axis refers to regional development, while the vertical axis concerns the innovativeness of the given region. This visual interpretation can be seen in Fig 6. The more developed regions are located on a Pareto chart.

**Fig 7:**
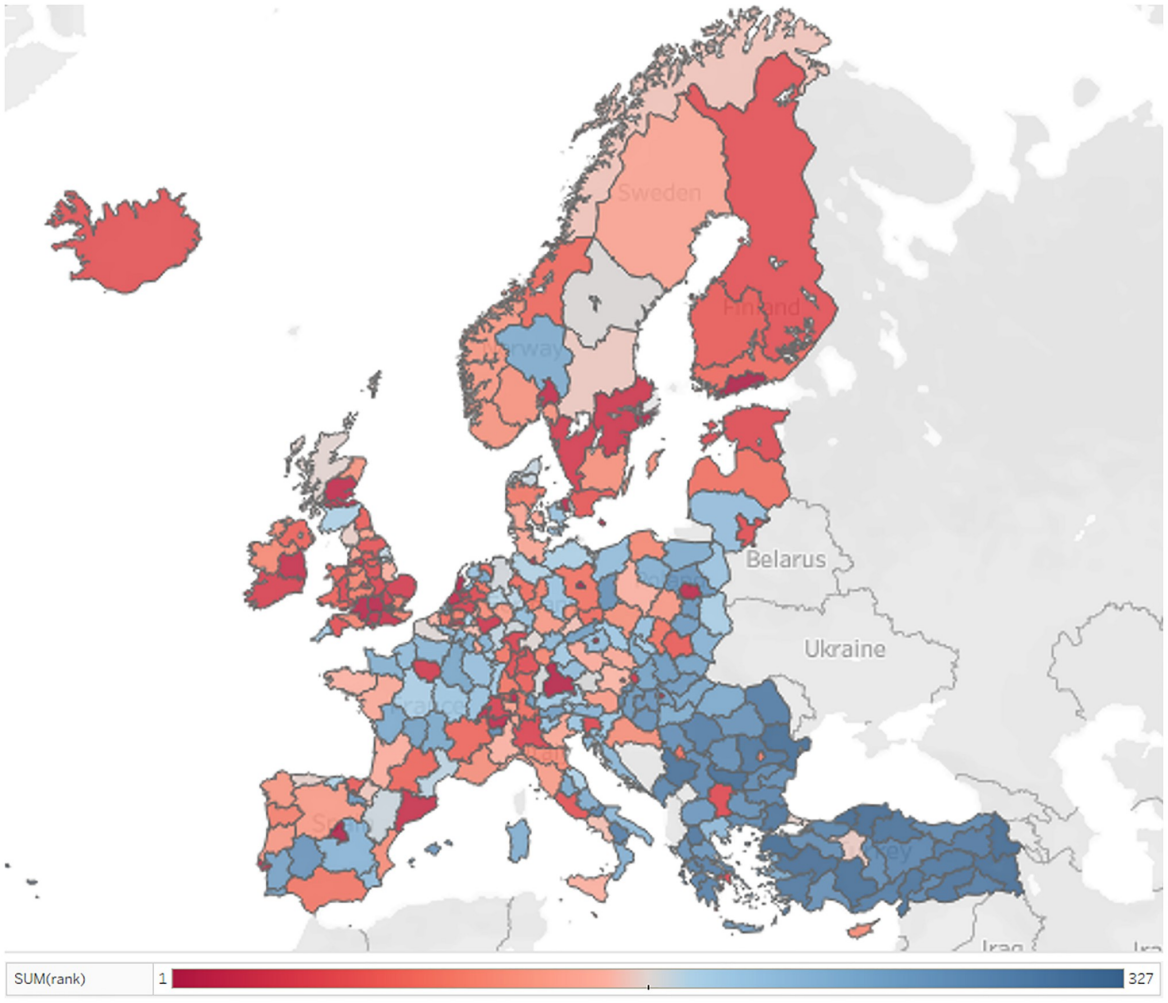
European NUTS 2 regional I4.0 readiness ranking based on the Promethee method [48].

The developed ranking is visualized in a map presented in Fig 7. According to the ranking, the most developed region is located in southern Finland, in the Helsinki-Uusimaa region to be exact, followed by the region of the capital city of the Czech Republic, that is, Prague and of Germany, namely Berlin.

### The correlation of the I4.0+ index with economic, innovation and competitiveness indexes

In this section, in order to provide a realistic and more in-depth picture, we compare our index with economic-related indicators, and existing indexes which aimed to rank regions according to innovation capability and competitiveness.

**Fig 8:**
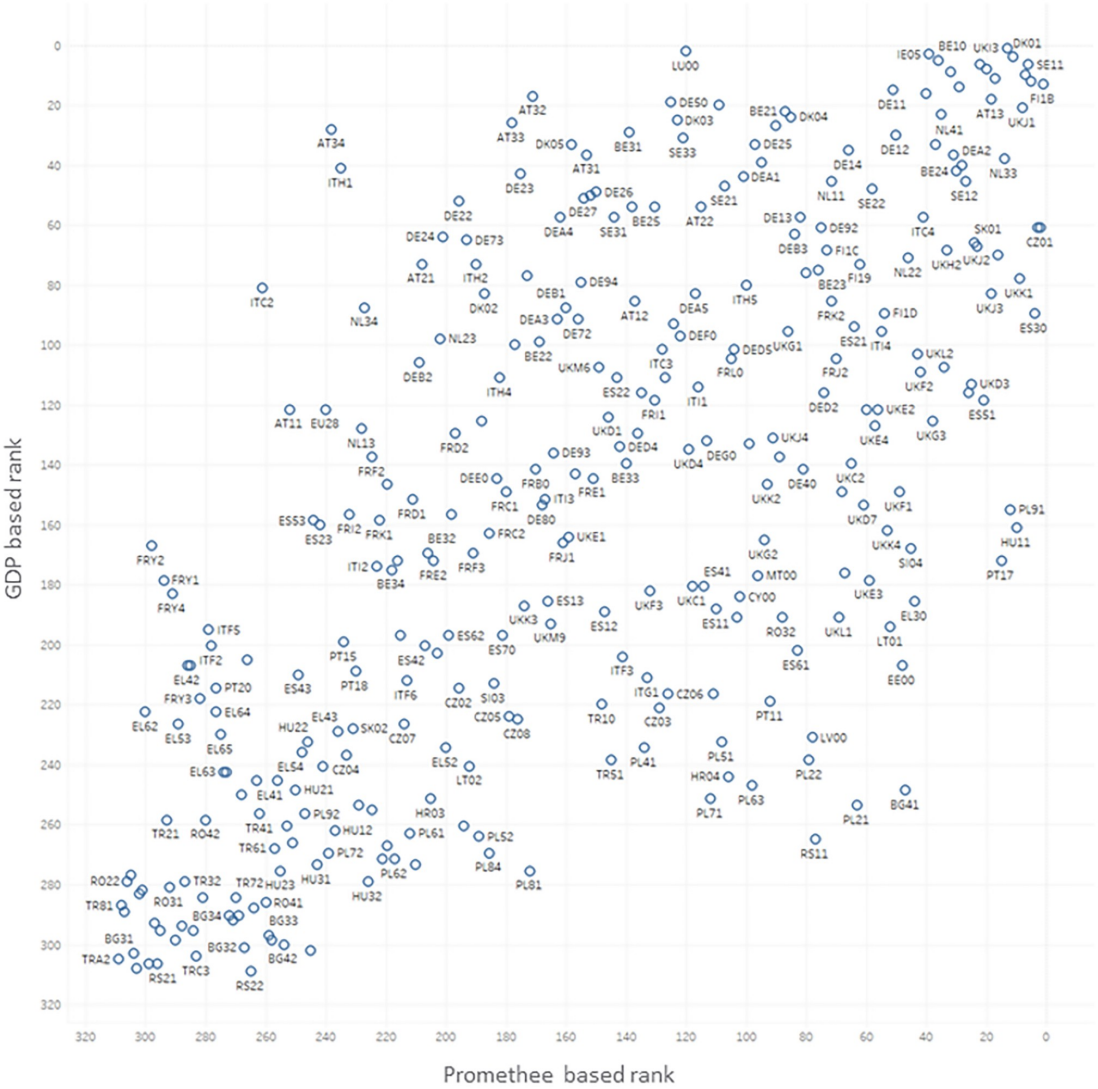
Correlation between GDP and our I4.0+ ranking system (r^2^ = 0.68).

**Fig 9:**
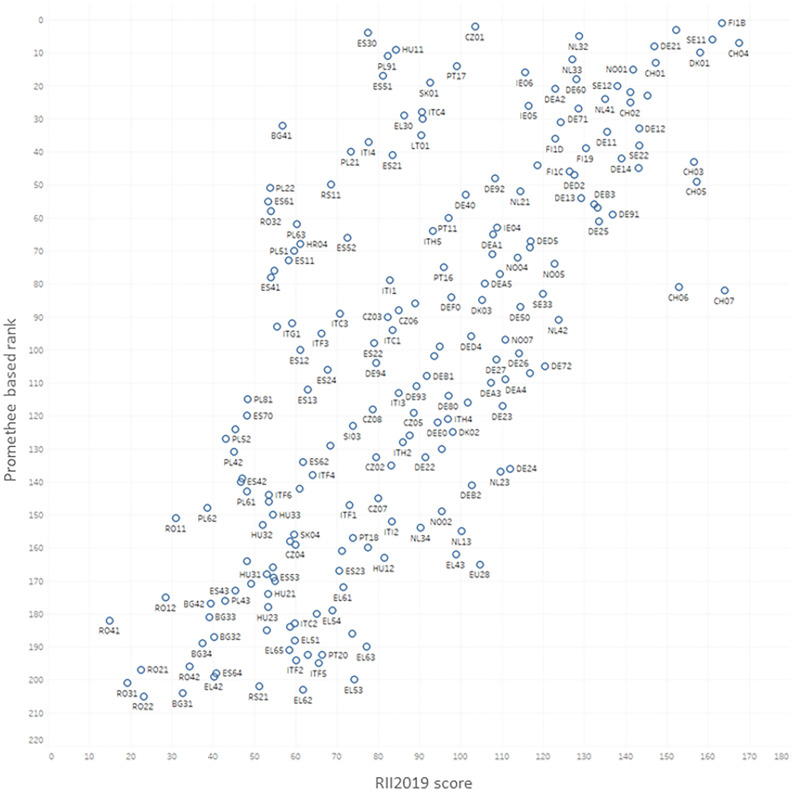
Correlation between the proposed I4.0+ ranking index and the Regional Innovation Index (RII) (r^2^ = 0.70).

1. Comparing the I4.0+ index with GDP is essential to identify the relationship between European regional economic growth and the regional I4.0 ranking based on the Promethee method. The results indicate a correlation of 0.68, which proves the connection between economic development and our proposed methodology.
  It should be noted that other factors influence GDP as well so, in this regard, it is hard to identify the developing effect ofI4.0 on economic growth. Fig 8 underlines the fact that there are regions with relatively high GDPs compared to our index, as their economic growth originates from factors other than the I4.0-related field.
2. The relationship between regional development (Real growth rate of regional gross value added (GVA)) and the proposed innovation index reflects that regions that are more backward according to our ranking can improve without applying the I4.0 concept by investing in the future. Therefore, those regions who do not take further steps to improve their overall economic prosperity are unable to emerge.
3. According to the Regional Innovation Scoreboard 2019 (RIS) report, its composite indicator Regional Innovation Index (RII), which is used to measure the average innovation performance, is also correlated with the proposed index.
Fig 9 shows a correlation of 0.70, which clearly represents the similarity. The horizontal axis refers to the innovation feature, while the vertical one concerns the specific features of I4.0. It nicely represents that for regions considered to be moderate innovators, in order to advance to the next level, the governmental focus is firmly on the development of innovation and the application of the I4.0 concept. These regions are located above the diagonal.
4. The Regional Competitiveness Index is also a core indicator that needs to be correlated, as competitiveness provides a stable foundation of which to apply the I4.0 concept. By comparing the two indexes, a correlation of 0.70 was calculated. It is worth noting that there are regions where I4.0 is not a priority, even though they still belong to the competitive regions such as regions of AT and LU.

In conclusion, the proposed I4.0+ index can determine regional development and innovation capability (can be one of its factors).

## Conclusions

This paper aims to provide an accessible tool for strategic planning and monitoring that regional stakeholders can utilize. The created concept can facilitate the understanding of the current status of regions according to their I4.0 relation and can contribute to elaborate medium-term development roadmap. For these reasons, a regional I4.0-specific indicator system was created, which makes the concept measurable and applicable. Indicators are categorized into five groups that support areas in favour of regional I4.0 readiness namely higher education and lifelong learning, the labour market, innovation, investment and technology. All segments have their specific scope, although in the Triple Helix, the actors/segments amplify their effect and increase the development and innovative features of regions by cross-collaborating actions. According to the spatio-temporal coverage, the desired data availability is the NUTS 2 regional level, and as recent as possible. Bearing in mind the purpose of measuring I4.0 readiness, the selection of indicators was strongly dependent on their specific relation to the area, e.g. I4.0-related publications, patents or educational training appearances. Moreover, the appearance of I4.0-related news stories was measured at the regional level and used as a “proxy” indicator to identify the regional connection to the field.

An I4.0+ composite indicator was generated from the aforementioned group of indicators and applied to the regions of the European Union. The importance of the employment factor and innovation activities as highlighted according to both the SRD and Promethee methods of ranking regions. The most advanced region is the Helsinki-Uusimaa region in Finland, followed by the capital city of the Czech Republic, that is Prague and of Germany, namely Berlin region.

The relevance of the index emphasized by the results of the correlations with other indicators. With both economic growth (GDP, Change of GDP) and innovation (RII), competitiveness (RCI) indexes approximates to correlation of 0.7. This similarity refers to the I4.0 fostering effect of innovation, competitiveness and economic development. We also revealed the untapped potential with regard to the application of the index as it can function as (1) a strategic governmental tool to stimulate the regional economy, (2) a decision-making tool of territorial councils to define in which field they should invest, (3) “heat map” for investors to determine which region has potential in terms of development and a stable economic environment, and (4) a tool for entrepreneurs to qualify the capability of regions to adapt to changes and remain competitive.

## Supporting information

S1 Appendix Regional indicator systems characteristics and their applicability. This file provides an overview of regional indicators.
(XLSX)

S2 Appendix Overview of Industry 4.0 readiness models. This file provides an overview of Industry 4.0 readiness models.
(XLSX)

S3 Appendix Data describing the regional Industry 4.0 readiness index. This file provides the description of variables and all the supporting data is available in the following repository link: http://dx.doi.org/10.17632/23gwn43ygp.1.
(XLSX)
